# Adverse Effects of Direct Acting Antivirals in HIV/HCV Coinfected Patients: A 4-Year Experience in Miami, Florida

**DOI:** 10.3390/diseases6020051

**Published:** 2018-06-19

**Authors:** Jose Armando Gonzales Zamora

**Affiliations:** Division of Infectious Diseases, Department of Medicine, University of Miami. Miller School of Medicine, Miami, FL 33136, USA; jxg1416@med.miami.edu or jgonzales2010@hotmail.com; Tel.: +1-706-284-3510; Fax: +1-305-243-4037

**Keywords:** adverse effects, Hepatitis C, HIV, direct acting antivirals

## Abstract

Introduction: The new direct acting antivirals (DAA) have demonstrated low rates of adverse effects in controlled studies. However, real world-studies have disclosed emerging toxicities and drug-drug interactions in special populations. Methods: We conducted a retrospective review of HIV/HCV coinfected patients who were treated with DAA at Jackson Memorial Hospital from 2014 to 2017. Our aim was to determine the adverse effects (AE) and factors that are associated with AE in HIV/HCV individuals who are treated with DAA. Results: There were 78 coinfected patients treated with DAA. AE that were secondary to DAA were reported by 21 (26.9%) patients. The most common AE were fatigue (47.6%), gastrointestinal symptoms (38.1%), anemia (14.3%), and headache (14.3%). In comparison with the rest of the study cohort, the patients who developed AE were more often Caucasian (33.3% vs. 10.5%, *p* = 0.017) and were more frequently treated with PrOD/Ribavirin (9.5% vs. 0%, *p* = 0.018). In terms of antiretroviral therapy (ART), there was a trend towards a more frequent use of TDF/FTC + NNRTI (33.3% vs. 14%, *p* = 0.055). Conclusions: These findings demonstrated good tolerability of DAAs in HIV/HCV coinfected patients. More real-world studies are needed to explore the variables that are associated with AE.

## 1. Introduction

The new direct acting antivirals (DAA) have revolutionized the treatment of hepatitis C, providing cure rates of 95–99% and showing a much better safety profile in comparison to old interferon-based regimens [1]. Low rates of adverse effects that are secondary to DAA have been reported in controlled studies; however, real world-studies have disclosed emerging toxicities and drug-drug interactions in special populations [2,3]. There is also very limited information about the factors that are associated with the adverse effects (AE) of DAA in HIV/HCV cohorts. Here, we contribute with data about the tolerability of DAA in 4 years of experience treating HIV/HCV coinfected patients in our clinical practice.

## 2. Material and Methods

We conducted a retrospective review of HIV/HCV coinfected patients that received treatment with DAA at the Ryan White Clinic of Jackson Memorial Hospital in Miami, Florida, USA. Our aim was to evaluate the adverse effects that are secondary to DAA in HIV/HCV coinfected patients from January 2014 to December 2017, and to identify the factors associated with the DAA adverse effects.

We adopted the definitions of adverse effects of the U.S. Food and Drug Administration. An adverse effect was defined as any untoward medical occurrence associated with the use of a drug in humans, whether or not it was considered drug-related. Severe adverse effects were defined as death, any life-threatening event, hospital admission, prolonged hospitalization, persistent or significant incapacity or substantial disruption of the ability to conduct normal life functions, congenital anomaly/birth defect, or any event considered serious based upon appropriate medical judgement [4]. For this study, we included all of the population of HIV/HCV coinfected patients treated with DAA in our clinic (78 patients). Clinical records were reviewed in order to collect demographic, clinical, laboratory, and treatment data. Patients with AE were compared to the rest of the study cohort using Chi-square for categorical variables and t-Student for continuous variables. All tests were 2-tailed, and a *p* value < 0.05 was considered statistically significant. The odds ratio (OR) was calculated with a 95% confidence interval (CI). SPSS version 22 statistical software (IBM Corp, Armonk, NY, USA) was used for analysis.

## 3. Results

There were 78 HIV/HCV coinfected patients treated with DAA, of which 25 (32.1%) were females and 53 (67.9%) were males, with a mean age of 55.6 (SD ± 7.88) years. The majority of patients were African American (57.7%). Most patients (98.6%) had undetectable HIV viral load, and the mean CD4 count was 637.68 cells/uL (SD ± 334.35). Antiretroviral therapy was received by 96.2% of patients. Tenofovir disoproxil fumarate (TDF)/Emtricitabine (FTC) plus protease inhibitor (26.92%), TDF/FTC plus integrase inhibitor (19.23%), and TDF/FTC plus non-nucleoside reverse transcriptase inhibitor (19.23%) were the most common antiretroviral regimens. HCV Genotype 1a was the most prevalent (61%). Advanced liver disease and cirrhosis were found in 28 (35.89%) and 12 (15.4%) patients, respectively. Most individuals were treated with Ledipasvir/Sofosbuvir (71.8%) and Simeprevir/Sofosbuvir (15.4%). Other regimens that were used were Elbasvir/Grazoprevir, Paritaprevir/ritonavir/ombitasvir/dasabuvir (PROD) plus ribavirin, and Ledipasvir/Sofosbuvir plus ribavirin, with two patients in each case. PROD and Sofosbuvir/Velpatasvir were received by 1 patient in both cases. The overall rate of sustained virologic response at 12 weeks post-treatment (SVR12) was 82.1% (Table 1).

**Figure 1:**
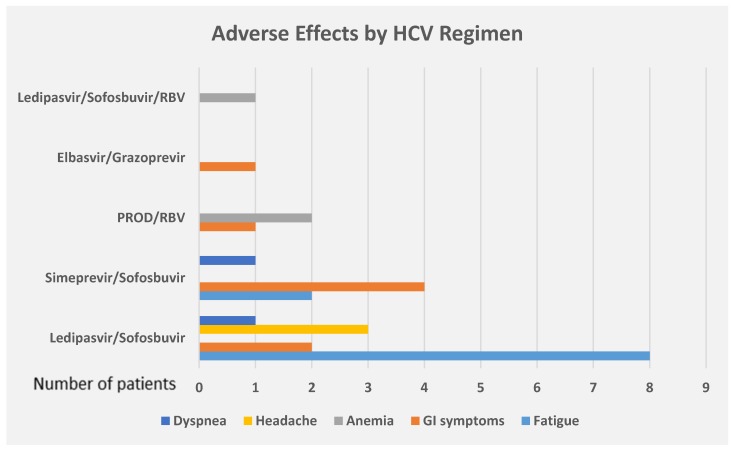
Adverse effects of DAA by the regimen of HCV treatment. PROD = Paritaprevir/ritonavir/ombitasvir/dasabuvir, RBV = Ribavirin, GI = Gastrointestinal.

Adverse effects that were secondary to DAA were reported by 21 (26.9%) patients. The most common AE were fatigue (47.6%), gastrointestinal symptoms (38.1%), anemia (14.3%), and headache (14.3%). Dyspnea was observed in only 2 patients. No serious AE were reported. No patients discontinued HCV treatment due to AE. Of the 56 patients who were treated with Ledipasvir/Sofosbuvir, 8 (14.3%) patients developed fatigue, 3 (5.4%) patients developed headache, 2 (3.6%) patients had gastrointestinal side effects, and 1 (1.8%) patient presented dyspnea. Simeprevir/sofosbuvir was received by 12 patients, of whom 4 (33.3%) developed gastrointestinal side effects, 2 (16.7%) developed fatigue, and 1 (8.3%) presented dyspnea. Only 2 patients were treated with Elbasvir/Grazoprevir and one of them developed gastrointestinal side effects. Of the 2 patients who were treated with PROD/Ribavirin, both developed anemia and one patient developed gastrointestinal side effects (Figure 1).

In comparison with the rest of the study cohort, the patients who developed AE were more often Caucasian (33.3% vs. 10.5%, *p* = 0.017) and were treated more frequently with PrOD/Ribavirin (9.5% vs. 0%, *p* = 0.018). In terms of antiretroviral therapy (ART), there was a trend towards a more frequent use of TDF/FTC + NNRTI (33.3% vs. 14%, *p* = 0.055). The higher frequency of AE did not correlate with hemoglobin, transaminases, total bilirubin, albumin, platelet count, CD4 count, cure rates, or degree of liver fibrosis (Table 1).

## 4. Discussion

The overall rate of adverse effects in our study was 26.9%, which is much lower than those reported in other real-world studies. The study conducted by Hawkins et al. on HIV/HCV coinfected patients revealed that 48% of patients experienced at least one adverse event, which is similar to the findings reported by Bruno et al., who found a rate of 59.7% [5,6]. Regarding severe adverse effects, the frequency ranges from 0% to 3.2% [6]. We did not observe any severe adverse effects in our population. Additionally, treatment discontinuation that was secondary to adverse effects was not seen, which suggests good tolerability of DAA in HIV/HCV coinfected patients. This finding was in concordance to other studies, in which the development of side effects did not lead to treatment interruptions or discontinuations [5,7].

We observed a predominant use of Sofosbuvir/Ledipasvir in our center. In this group of patients, the most common adverse effects were fatigue, headache, and gastrointestinal symptoms. These findings were similar to the ones reported in controlled studies [8]. In our cohort, fatigue was observed in 14.3% of patients on Sofosbuvir/Ledipasvir, which stands within the range that is reported in clinical trials [9]. Regarding headache, we have found a rate of 5.4%, which is lower than the rate described in the literature (11–17%). Another important difference was the development of gastrointestinal symptoms. Our study showed a rate of 3.6%, which was lower than the frequency that was noted in controlled studies (10–16%) [8]. It is worth mentioning that newly diagnosed or worsening pulmonary arterial hypertension have been reported in case-series; however, it is difficult to establish true causality [2]. Cases of lactic acidosis have also been described, and the risk seems to increase in patients with severe liver disease [3]. No cases of pulmonary hypertension or lactic acidosis were reported in our study.

Simeprevir/sofosbuvir was the second most frequent DAA regimen used in our cohort. In the patients who were treated with this regimen, gastrointestinal symptoms were the leading adverse effects, reported in 33.3% of cases. Fatigue and dyspnea developed in 16.7% and 8.3% of patients, respectively. These findings are similar to those disclosed in clinical trials, in which fatigue and nausea were two of the most common side effects [9]. Dyspnea is reported infrequently in controlled and real-world studies, with a rate that can be as high as 4%, which is lower than the rate found in our patients (8.3%) [10]. A relatively common adverse effect seen in controlled studies is headache, which occurs in approximately 20% of patients on Simeprevir/sofosbuvir; however, none of our patients developed headaches during their treatment with this regimen [9]. Other clinically significant adverse effects that are reported in the literature are pruritus (14%), rash (16%), and photosensitivity (5%) [9,11]. None of these side effects was reported in our study cohort.

In terms of treatment adherence, while some real-world studies have identified low adherence to antiretroviral therapy in HIV-infected patients, poor compliance to DAA has not been described as a limiting factor in the treatment of HCV in HIV/HCV coinfected patients [12]. We have found good compliance with HCV treatment in our clinical practice, with a treatment completion rate of 93.6%. Only four patients were lost to follow-up (5.1%), and one patient did not finish his treatment due to other reasons (a motor vehicle accident). We had 6 patients who completed their treatment but did not have laboratory studies at 12 weeks post treatment to assess for cure. This limitation influenced the overall HCV cure rate or SVR12 reported in our center, which was 82.1%—a percentage lower than those reported in controlled and recent real world-studies [5].

Studies comparing DAA adverse effects between HCV monoinfected and HIV/HCV coinfected patients are very limited and have shown contradictory results. The study conducted by Bruno et al. disclosed a higher rate of adverse effects in HIV/HCV patients when compared with HCV monoinfected patients (59.7% vs. 57.2%, *p* = 0.03) [6]. Fatigue was observed in 17% of coinfected patients and 10% of monoinfected patients—a difference that achieved statistical significance (*p* = 0.003). Jaundice was also identified as an adverse effect that was more frequently developed by HIV/HCV coinfected individuals (9.7% vs. 1.3%, *p* < 0.0001) [6]. On the other hand, a study aimed to evaluate the safety and effectiveness of sofosbuvir/simeprevir reported a more frequent development of adverse effects in HCV monoinfected patients when compared with HIV/HCV coinfected individuals (54.2% vs. 51.7%, *p* = 0.04) [13]. Our center is exclusively oriented to the treatment of HIV-infected individuals; therefore, comparison with HCV monoinfected patients was not feasible. 

Regarding factors associated with adverse effects, we have found a higher frequency of Caucasian ethnicity in patients that developed these events. There are no reports of this association in the literature. We evaluated the DAA regimen of these patients, looking for an association, and we did not find any predominant regimen that could explain this finding. Although our population was small, we believe ethnicity is a factor that should be explored in bigger studies. In terms of the DAA regimen, we found a higher frequency of PROD/ribavirin use in patients that developed adverse effects. Only two patients received this regimen in our study, making it very difficult to establish a real association; however, we observed that both patients developed anemia. In our opinion, it was clear that ribavirin was the culprit, because anemia is a frequent side effect of this medication that can occur in up to 35% of patients [14,15]. The overall frequency of anemia in our study was low (3.8%) and was found exclusively in patients that received ribavirin as part of their treatment. It is worth mentioning that the only absolute indication to add ribavirin to a DAA regimen is decompensated cirrhosis, as stated by current guidelines [16]. In our study, two patients who received ribavirin-containing regimens were non-cirrhotic and one patient had compensated cirrhosis. Of note, these patients were treated in 2015, when the availability of second generation DAAs was still limited in our center and before the release of the new HCV treatment guidelines [16]. 

Our study also revealed a trend towards a more frequent use of TDF/FTC plus non-nucleoside reverse transcriptase inhibitor (NNRTI) in patients with adverse effects (*p* = 0.055). Of the patients treated with TDF/FTC plus NNRTI, three were treated with ledipasvir/sofosbuvir, three were treated with simeprevir/sofosbuvir, and one was treated with Elbasvir/Grazoprevir. When treating patients with HIV/HCV coinfection, one of the main parameters to assess is drug-drug interaction. Several pharmacokinetic studies have detected higher levels of TDF in patients treated with ledipasvir, which could potentially increase the risk of nephrotoxicity [17]. In our study, we did not observe any cases of acute renal failure, and the higher rate of adverse effects was only seen when TDF was given with NNRTI. We did not identify any significant difference when TDF was used with protease or integrase inhibitors. In terms of interactions between simeprevir and NNRTI, this DAA is contraindicated in patients receiving efavirenz, nevirapine, or etravirine [18]. However, no drug-drug interactions are expected with rilpivirine, which is the drug used in our study. We did not observe any concomitant use of contraindicated NNRTIs with simeprevir in our patients. Other factors besides drug-drug interaction could have potentially played a role in the higher frequency of adverse effects with the concomitant use of TDF/FTC and NNRTI.

## 5. Conclusions

These findings demonstrated good tolerability of DAAs in HIV/HCV coinfected patients who were treated in our practice. White race, treatment with PrOD/Ribavirin, and possibly the concomitant use of TDF/FTC plus NNRTI were variables that were associated with DAA adverse effects. More real-world studies are needed to explore these possible associations.

## Figures and Tables

**Table 1:** Clinical, laboratory, and treatment characteristics of HIV/HCV coinfected patients and a comparison by the development of DAA adverse effects.

Variable	HIV/HCV Patients	No Adverse Effects	Adverse Effects	*p*	OR (95% CI)
	(*n* = 78)	(*n* = 57)	(*n* = 21)		
Age	55.64 ± 7.88	55.19 ± 8.26	56.86 ± 6.77	0.41	-
Sex (male)	53 (67.9%)	41 (71.9%)	12 (57.1%)	0.22	0.63 (0.31–1.29)
Race					
(a) Black	45 (57.7%)	35 (61.4%)	10 (47.6%)	0.27	0.67 (0.32–1.38)
(b) White	13 (16.7%)	6 (10.5%)	7 (33.3%)	**0.017**	2.5 (1.26–4.96)
(c) Hispanic	20 (25.6%)	16 (28.1%)	4 (19.0%)	0.42	0.68 (0.26–1.79)
HAART	75 (96.2%)	56 (98.2%)	19 (90.5%)	0.11	0.38 (0.16–0.93)
Hemoglobin	13.72 ± 1.63	13.8 ± 1.56	13.54 ± 1.82	0.53	-
AST	74.44 ± 67.471	78.12 ± 76.35	64.43 ± 32.55)	0.43	-
ALT	77.46 ± 82.57	83.39 ± 94.77	61.38 ± 26.96	0.3	-
Albumin	4.113 ±0.63	4.18 ± 0.63	3.94 ± 0.60	0.14	-
Total bilirubin	0.96 ± 0.92	0.99 ± 0.94	0.90 ± 0.89	0.72	-
Platelet count	188.92 ± 70.18	188.72 ± 69.60	189.48 ± 73.45	0.97	-
CD4 count	637.68 ± 334.35	610.54 ± 312.63	711.33 ± 386.73	0.24	-
CD4/CD8	0.92 ± 0.63	0.92 ± 0.625	0.94 ± 0.65	0.87	-
CD4%	30.14 ± 11.26	29.65 ± 11.54	31.48 ± 10.59	0.53	-
CD4 count < 500	29 (37.2%)	22 (38.6%)	7 (33.3%)	0.94	0.85 (0.39–1.85)
ART regimen					
(a) TDF/FTC + NNRTI	15 (19.2%)	8 (14.0%)	7 (33.3%)	0.055	2.10 (1.03–4.28)
(b) TDF/FTC + PI	21 (26.9%)	17 (29.8%)	4 (19.0%)	0.34	0.64 (0.24–1.68)
(c) TDF/FTC + InSTI	15 (19.2%)	12 (21.1%)	3 (14.3%)	0.5	0.70 (0.24–2.07)
(d) TAF + InSTI	4 (5.1%)	3 (5.3%)	1 (4.8%)	0.93	0.93 (0.16–5.26)
(e) ABC/3TC + InSTI	7 (9.0%)	6 (10.5%)	1 (4.8%)	0.43	0.51 (0.08–3.23)
(f) ABC/3TC + PI	4 (5.1%)	3 (5.3%)	1 (4.8%)	0.93	0.93 (0.16–5.26)
(g) Other regimens	9 (11.5%)	7 (12.3%)	2 (9.5%)	0.74	0.81 (0.22–2.91)
Prior Tx with IFN	22 (28.2%)	15 (26.3%)	7 (33.3%)	0.54	1.27 (0.59–2.73)
Liver biopsy	33 (42.3%)	22 (38.6%)	11 (52.4%)	0.27	1.50 (0.72–3.11)
Elastography	15 (19.2%)	13(22.8%)	2 (9.5%)	0.19	0.44 (0.12–1.70)
Genotype					
(a) 1a	47 (61.0%)	34 (59.6%)	13 (61.9%)	0.86	1.07 (0.50–2.23)
(b) 1b	25 (32.5%)	18 (31.6%)	7 (33.3%)	0.88	1.06 (0.49–2.30)
(c) Others	6 (7.7%)	5(8.8%)	1(4.8%)	0.56	0.60 (0.09–3.73)
HCV10log	6.18 ± 0.76	6.137 ± 0.82	6.30 ± 0.56	0.4	-
Creatinine	1.05 ± 0.38	1.09 ± 0.41	0.95 ± 0.24	0.14	-
Advanced liver disease (F3, F4)	28 (35.9%)	20 (35.1%)	8 (38.1%)	0.81	1.10 (0.52–2.33)
Cirrhosis	12 (15.4%)	7 (12.3%)	5 (23.8%)	0.21	1.72 (0.78–3.80)
HCV treatment					
(a) Ledipasvir/Sofosbuvir	56 (71.8%)	44 (77.2%)	12 (57.1%)	0.08	0.52 (0.26–1.07)
(b) Simeprevir/Sofosbuvir	12 (15.4%)	7 (12.3%)	5 (23.8%)	0.21	1.72 (0.78–3.80)
(c) PROD/RBV	2 (2.6%)	0 (0%)	2 (9.5%)	**0.018**	4.00 (2.71–5.90)
(d) Elbasvir/Grazoprevir	2 (2.6%)	1 (1.8%)	1 (4.8%)	0.46	1.90 (0.45–7.99)
(e) Ledipasvir/Sofosbuvir + RBV	2 (2.6%)	1 (1.8%)	1 (4.8%)	0.46	1.90 (0.45–7.99)
(f) Sofosbuvir + RBV	2 (2.6%)	2 (3.5%)	0 (0%)	0.39	-
(g) PROD	1 (1.3%)	1 (1.8%)	0 (0%)	0.54	-
(h) Sofosbuvir/Velpatasvir	1 (1.3%)	1 (1.8%)	0 (0%)	0.54	-
Tx duration (12 weeks)	71 (91.0%)	53 (98.1%)	18 (90.0%)	0.11	0.38 (0.16–0.93)
SVR12 (ITT)	64 (82.1%)	44 (77.2%)	20 (95.2%)	0.07	4.38 (0.64–29.94)
Completed HCV Tx	73 (93.6%)	54 (94.7%)	19 (90.5%)	0.5	0.65 (0.21–2.03)
Lost to follow-up	4 (5.1%)	3 (5.3%)	1 (4.8%)	0.93	0.93 (0.16–5.26)

ART = antiretroviral therapy, AST = aspartate aminotransferase, ALT = alanine aminotransferase, TDF = tenofovir disoproxil fumarate, FTC = emtricitabine, NNRTI = non-nucleoside reverse transcriptase inhibitor, PI = protease inhibitor, InSTI = Integrase inhibitor, ABC = abacavir, 3TC = lamivudine, Tx = treatment, IFN = interferon, PROD = Paritaprevir/ritonavir/ombitasvir/dasabuvir, RBV = ribavirin, SVR12 = sustained virologic response at 12 weeks post-treatment, ITT = intention to treat. The *p* values that achieved statistical significance (*p* < 0.05) are in bold.
